# Single-center perioperative management and 1-year heart transplantation outcomes for patients with adult congenital heart disease

**DOI:** 10.1016/j.jhlto.2025.100243

**Published:** 2025-03-07

**Authors:** Reice Robinson, Samvel Gaboyan, Laith Alshawabkeh, Ryan Reeves, Eugene Golts, Victor Pretorius, John Nigro, Mark Kearns, Howaida El-Said, Matthew Carazo, Kimberly Hong, Eric Adler, Paul J. Kim, Amanda Topik, Deborah Raleigh, Angela Meier, Sarah Ellis, Ramon Sanchez, Kamyar Afshar, Aarya Kafi, Christine Lin, Gordon Yung, Aleah Brubaker, Gabriel Schnickel, Kristin Mekeel, Jennifer Berumen, Justin Parekh, Pranab Barman, Veeral Ajmera, Yuko Kono, Irine Vodkin, Marcus A. Urey

**Affiliations:** a Department of Medicine, University of California, San Diego, San Diego, California; b Department of Medicine, Division of Pulmonary, Critical Care, and Sleep Medicine & Physiology, University of California, San Diego, San Diego, California; c Department of Medicine, Division of Cardiology, University of California, San Diego, San Diego, California; d Department of Surgery, Division of Cardiothoracic Surgery, University of California, San Diego, San Diego, California; e Department of Surgery, Division of Cardiac Surgery and Cardiac Transplant Services, Rady Children's Hospital, San Diego, California; f Department of Medicine, Division of Cardiology, Rady Children's Hospital, San Diego, California; g Department of Anesthesiology, University of California, San Diego, San Diego, California; h Department of Surgery, Division of Transplant and Hepatobiliary Surgery, University of California, San Diego, San Diego, California; i Department of Medicine, Division of Gastroenterology and Hepatology, University of California, San Diego, San Diego, California

**Keywords:** adult congenital heart disease, heart transplantation, multiorgan transplantation, Fontan, aortopulmonary collateral vessels

## Abstract

**Background:**

Additional data are needed to define the optimal listing strategy and peri-transplant management for adults with congenital heart disease (ACHD). In this study, we evaluated the perioperative management and 1-year outcomes for 30 patients who underwent orthotopic heart transplantation (OHT) for ACHD.

**Methods:**

We conducted a single-center retrospective case series of all patients who received an OHT at our institution for ACHD from January 1, 2017 through June 30, 2024. Descriptive statistical analyses were used to illustrate the baseline characteristics, peri-transplant management, and 1-year outcomes of participants.

**Results:**

Seventeen (56.7%) patients received a heart-only transplant, 4 (13.3%) had a heart-lung transplant, 8 (26.7%) had a heart-liver transplant, and 1 (3.3%) had a heart-liver-kidney transplant. Embolization of aortopulmonary and venous collateral vessels before transplantation was performed in 12 (80%) Fontan patients, with a median of 2 procedures per patient. Twenty-eight (93.3%, *n* = 30) patients were alive at 90 days, and 26 (92.9%, *n* = 28) were alive at 1 year.

**Conclusions:**

This cohort of ACHD patients had a 92.9% 1-year survival rate, consistent with other high-volume centers in the United States. In this paper, we discuss our institutional practices that address barriers to transplant for ACHD patients, such as early referral for transplant, indications for listing, pretransplant embolization of collateral vessels, and multiorgan transplant.

## Background

The prevalence of adults with congenital heart disease (ACHD), particularly those with severe congenital heart disease (CHD), now outnumbers the prevalence of children with CHD.^1^ As they age, ACHD patients commonly experience heart failure; therefore, there is an increased number of ACHD patients who are referred for heart transplant evaluation.^2^ ACHD patients undergoing orthotopic heart transplantation (OHT) have higher waitlist mortality, higher postoperative mortality, and more frequent post-transplantation complications compared to non-CHD transplanted patients.^3^, ^4^ However, ACHD patients have equivalent, if not better, long-term survival compared to other adult OHT patients.^3^, ^5^, ^6^, ^7^

Historically, ACHD patients have had many barriers to transplantation. There is no consensus on the proper time for ACHD patients to be listed for transplant due to the changing patient population and limited published data. However, their higher risk of death on the waitlist suggests ACHD patients should be considered earlier for transplant than is current practice and are likely disadvantaged by current allocation policies.^8^

The current allocation system prioritizes temporary mechanical circulatory support (MCS) and inotropes, which can be considered in this patient population.^2^ Historically, the use of durable MCS has been limited to those with acquired heart disease and an anatomically normal heart, disadvantaging ACHD patients on the waitlist.^2^ Recently, left ventricular assist devices and the total artificial heart have been successfully implanted in those with the failing Fontan, expanding the therapies available for this at-risk population.^9^, ^10^ While both temporary and durable MCS can be considered for ACHD patients, this technology remains underutilized thus highlighting the importance of waitlist position for ACHD patients. ACHD patients are often listed by “exception” which raises their position on the waitlist despite not meeting the clinical criteria for the stage.^2^

The complex anatomy of patients with CHD can cause multiorgan dysfunction, resulting in the need for multiorgan transplants, especially heart-lung and heart-liver transplants. Approximately 16% of heart transplants for ACHD in the United States are multiorgan transplants, and these patients have a higher 5 year mortality compared to heart-only transplants.^11^ Upon further analysis, this difference was entirely driven by patients who underwent heart-lung transplants with no difference for heart-kidney or heart-liver transplants.^11^ However, there is no difference in outcomes between ACHD and non-ACHD heart-lung transplant recipients, so this should not preclude ACHD patients from receiving a multiorgan transplant if needed.

Given the increasing number of ACHD patients progressing to transplant, with a high proportion needing multiorgan transplant, additional data are needed to help define the optimal listing strategy and peri-transplant management for ACHD patients. In this single-center, retrospective observational cohort study, we evaluated the perioperative management and 1-year outcomes for 30 consecutive patients who underwent transplantation for ACHD at our institution, including 13 patients who underwent multiorgan transplant.

## Materials and methods

We conducted a single-center retrospective case series of all patients who received an OHT at the University of California San Diego for ACHD from January 1, 2017 through June 30, 2024. Recipients of multiorgan transplants for ACHD were included. All included participants were undergoing their first OHT for congenital heart disease. Patients undergoing retransplant were excluded. Institutional review board exemption was granted (#803384). This study adheres to the principles of the Declaration of Helsinki formulated by the World Medical Association, the Declaration of Istanbul, and the International Society for Heart and Lung Transplantation Statement on Transplant Ethics. Informed consent was waived.

Data were collected from the electronic medical record by 1 reviewer. The primary outcome was 90-day and 1-year all-cause mortality. Secondary outcomes included status at listing, time from listing to transplant, ICU and hospital length of stay, episodes of rejection, and transplanted organ function at 1 year.

Descriptive statistical analyses were used to determine the baseline characteristics of participants. Categorical variables were expressed as frequency and percentage. Non-normally distributed variables were expressed as median and interquartile range (IQR). All statistical analyses were done using Stata software (version 18.5; StataCorp), and Excel software (version 16; Microsoft) was used for data reprocessing.

## Results

### Baseline characteristics

Thirty patients were included in the study, and 1 patient was excluded due to being a retransplant. Two patients died on the waitlist during this period. One patient was delisted for clinical deterioration and died soon thereafter. The other had a 6-month delay in listing due to external factors and subsequently suffered sudden cardiac death while listed. Baseline demographics of included patients are reported in Table 1. The median age at transplant was 31.5 years (*n* = 30, IQR 24-39). Fifteen (50.0%) patients had previously undergone a Fontan procedure, and 4 (13.3%) had an atrial switch. Seventeen (56.7%) received a heart-only transplant, 4 (13.3%) had a heart-lung transplant, 8 (26.7%) had a heart-liver transplant, and 1 (3.3%) had a heart-liver-kidney transplant. Five (16.7%) received a donation after circulatory death (DCD). Of these 5, 1 was a heart-liver-kidney transplant. The remaining 4 received a heart-only transplant. Five patients (16.7%) had a panel reactive antibody greater than 25%. Two patients (6.7%) had MCS (both left ventricular assist devices) before transplant.

**Table 1:** Baseline Demographics of Patients Transplanted for Adult Congenital Heart Disease

Characteristic	N = 30
Age, years	31.5 (24.0-39.0)
Panel reactive antibody, by quartile (%)	
0-25	25 (83.3%)
25-50	1 (3.3%)
50-75	2 (6.7%)
75-100	2 (6.7%)
Congenital defect	
Single ventricle palliated to Fontan circulation	15 (50.0%)
Transposition of great arteries palliated with atrial switch	4 (13.3%)
Tetralogy of Fallot	2 (6.7%)
Other	12 (40.0%)
Presence of left ventricular assist device	2 (6.7%)
Heart-only transplant	17 (56.7%)
Heart-lung transplant	4 (13.3%)
Heart-liver transplant	8 (26.7%)
Heart-liver-kidney transplant	1 (3.3%)
Blood type	
A	9 (30.0%)
B	4 (13.3%)
AB	1 (3.3%)
O	16 (53.3%)
Heart transplant allocation score	
Before policy change in 2018, *n* = 8	
1A	5 (62.5%)
1B^a^	3 (37.5%)
2	0 (0.0%)
Exception	4 (50.0%)
After policy change in 2018, *n* = 22	
1	0 (0.0%)
2	3 (13.6%)
3	0 (0.0%)
4	19 (86.4%)
5	0 (0.0%)
6	0 (0.0%)
Exception	3 (13.6%)
Lung allocation score, *n* = 4	36.0 (23.8-48.2)
Model for end-stage liver disease-sodium score, *n* = 7^b^	12.0 (7.0-17.0)
Creatinine levels pretransplant (mg/dl), *n* = 1^c^	2.68
Estimated glomerular filtration rate pretransplant (ml/min/1.73 m^2^), *n* = 1^c^	20

Values shown as frequency (percent) or median (interquartile range).

a One patient listed under pediatric list remained in that status when transplanted as an adult.

b Exclusion of 2 patients due to taking Warfarin.

c Only patient who received a kidney transplant (*n* = 1).

The heart allocation policy was changed in 2018. Among our patients who were transplanted before the change (*n* = 8), 5 (62.5%) were listed as status 1A, and 3 (37.5%) were listed as status 1B. Four (50.0%) were listed by exception. Twenty-two patients were listed after the change in 2018. Of these patients, 3 (13.6%) were listed as status 2, and 19 (86.4%) were listed as status 4. Three (13.6%) were listed by exception.

### Perioperative management

Our perioperative management is detailed in Table 2. Donation after brain death (DBD) accounted for 83.3% of transplants, and hearts were transferred via cold storage. DCD accounted for 16.7% of transplants, and hearts were transferred via cold storage if normothermic regional perfusion was used (3 of 5) and warm perfusion if the Organ Care System was used (2 of 5).

**Table 2:** Perioperative Management of Patients Transplanted for Adult Congenital Heart Disease

Characteristic	N = 30
Donation after circulatory death, *n* = 30	25 (83.7%)
Transported via cold storage, *n* = 25	25 (100%)
Transported via warm perfusion, *n* = 25	0 (0.0%)
Donation after brain death, *n* = 30	5 (16.7%)
Transported via cold storage, *n* = 5	3 (60.0%)
Transported via warm perfusion, *n* = 5	2 (40.0%)
Length of hospitalization post-transplant, days, *n* = 28^a^	30.0 (21.5-38.5)
Length of stay in ICU pre- and post-transplant, days, *n* = 28^a^	7.0 (5.0-9.0)
Induction, *n* = 30	
Thymoglobulin	13 (43.3%)
Basiliximab	5 (16.7%)
None	12 (40.0%)
Induction, heart-only, *n* = 17	
Thymoglobulin	9 (52.9%)
Basiliximab	1 (5.9%)
None	7 (41.2%)
Induction, heart-lung, *n* = 4	
Thymoglobulin	0 (0.0%)
Basiliximab	3 (75.0%)
None	1 (25.0%)
Induction, heart-liver, *n* = 8	
Thymoglobulin	4 (50.0%)
Basiliximab	1 (12.5%)
None	3 (37.5%)
Induction, heart-liver-kidney, *n* = 1	
Thymoglobulin	0 (0.0%)
Basiliximab	0 (0.0%)
None	1 (100.0%)
Need for dialysis pretransplant	0 (0.0%)
Need for dialysis post-transplant	7 (23.3%)
Need for vasopressors pretransplant	6 (20.0%)
Need for vasopressors post-transplant	30 (100.0%)
Need for vasopressors >14 days post-transplant, *n* = 28^a^	5 (17.9%)
Need for mechanical ventilation pretransplant	0 (0.0%)
Days on mechanical ventilation post-transplant, *n* = 28^a^	3.5 (2.0-5.0)
Need for extracorporeal membrane oxygenation (ECMO) pretransplant	1 (3.3%)
Need for ECMO post-transplant	5 (16.7%)
Initiated intraoperatively, *n* = 5	3 (60.0%)
Initiated postoperatively, *n* = 5	2 (40.0%)
Total days on ECMO post-transplant, *n* = 3^a^	8; 3 (100%)
Procedures to embolize collaterals pretransplant	2.0 (1.0-3.0)
Patients who underwent embolization of collaterals pretransplant, *n* = 15^b^	12 (80.0%)
Procedures to embolize collaterals post-transplant	0.0 (0.0-0.0)
Patients who underwent embolization of collaterals post-transplant, *n* = 15^b^	2 (13.3%)

Values shown as frequency (percent), median (interquartile range), or total sum with median (interquartile range).

a Exclusion of 2 patients due to deceased before initial discharge <14 days post-transplant.

b Only patients who underwent Fontan procedure.

Overall, 13 (43.3%) patients received induction with thymoglobulin, 5 (16.7%) with basiliximab, and 7 (28%) did not receive any induction. By transplant type, 9 (52.9%, *n* = 17) heart-only recipients received induction with thymoglobulin, compared to 0 (0.0%, *n* = 4) heart-lung and 4 (50.0%, *n* = 8) heart-liver. One (5.9%, *n* = 17) heart-only recipient received induction with basiliximab, compared to 3 (75.0%, *n* = 4) heart-lung and 1 (12.5%, *n* = 8) heart-liver. Seven (41.2%, *n* = 17) heart-only recipients received no induction, compared to 1 (25.0%, *n* = 4) heart-lung and 3 (37.5%, *n* = 8) heart-liver.

Among patients who had a Fontan, 12 (80.0%, *n* = 15) underwent embolization of aortopulmonary and venous collateral vessels before transplantation with a median of 2 (IQR 1-3, *n* = 15) procedures per patient. Post-transplantation, 2 (13.3%, *n* = 15) patients underwent embolization.

No patients required dialysis pretransplant, but 7 (23.3%) required renal replacement therapy afterward. Six (20.0%) patients required vasopressors pretransplant, and 30 (100%) required vasopressors afterward. Five (17.9%) patients continued to require vasopressors for more than 14 days. One (3.3%) patient required veno-arterial extracorporeal membrane oxygenation (VA-ECMO) pretransplant, and 5 (16.7%) required it postoperatively. Of the 5 patients on VA-ECMO post-transplant, 2 received VA-ECMO for left ventricular primary graft dysfunction, 1 for biventricular primary graft dysfunction, 1 for vasoplegia, and 1 remained on VA-ECMO after liver transplant although graft function appeared intact. Three (60%) patients had VA-ECMO initiated intraoperatively, and 2 (40%) had it initiated postoperatively. No patients required mechanical ventilation pretransplant, and the median number of days on the ventilator postoperatively was 3.5 (IQR 2.0-5.0). Postoperatively, the median intensive care unit (ICU) length of stay was 7 days (IQR 5.0-9.0), and hospital length of stay was 30 days (IQR 21.5-38.5).

### One-year outcomes

Table 3 reflects 1-year outcomes. Patients not yet at 1 year post-transplant were excluded. Twenty-eight (93.3%, *n* = 30) patients were alive at 90 days, and 26 (92.9%, *n* = 28) were alive at 1 year. There was no difference in 1-year mortality between patients who received a transplant from a DCD donor compared to DBD. Out of 28 patients, 3 (11.5%) patients experienced acute heart allograft rejection by 1 year, totaling 6 distinct episodes of rejection. Of the 4 patients with a heart-lung transplant, there was 1 episode of acute lung allograft rejection. Of the 7 patients with a heart-liver or heart-liver-kidney transplant, there was 1 episode of liver allograft rejection. Donor-specific antibody was transiently positive in 6 (23.1%) patients, absent in 20 (76.9%), and persistently positive in 0, *n* = 26. Three (13.6%, *n* = 22) patients developed cardiac allograft vasculopathy.

**Table 3:** One-Year Outcomes of Patients Transplanted for Adult Congenital Heart Disease

Characteristic	N = 30
Total episodes and patients with rejection, by organ	
Heart, *n* = 26^a^^,^^b^	6; 3 (11.5%)
Lung, *n* = 4	1; 1 (25.0%)
Liver, *n* = 7^c^^,^^d^	1; 1 (14.3%)
Total episodes and patients with heart rejection, by transplant type	
Heart-only, *n* = 15^c^^,^^d^	6; 3 (20.0%)
Heart-lung, *n* = 4	0; 0 (0.0%)
Heart-liver, *n* = 6^c^^,^^d^	0; 0 (0.0%)
Heart-liver-kidney, *n* = 1	0; 0 (0.0%)
Severity of heart rejection episodes, *n* = 6	
ACR 2R	4 (66.7%)
pAMR-2	2 (33.3%)
Cardiac allograft vasculopathy, *n* = 22	3 (13.6%)
Donor-specific antibody, *n* = 26	
Absent	20 (76.9%)
Transient	6 (23.1%)
Positive	0 (0.0%)
Living status at 90 days, by transplant type	
Heart-only, *n* = 17	16 (94.1%)
Heart-lung, *n* = 4	4 (100.0%)
Heart-liver, *n* = 8	7 (87.5%)
Heart-liver-kidney, *n* = 1	1 (100.0%)
Total, *n* = 30	28 (93.3%)
Living status at 1 year, by transplant type	
Heart-only, *n* = 16^d^	15 (93.8%)
Heart-lung, *n* = 4	4 (100.0%)
Heart-liver, *n* = 7^d^	6 (85.7%)
Heart-liver-kidney, *n* = 1	1 (100.0%)
Total, *n* = 28^b^	26 (92.9%)
Left ventricle ejection fraction (%), *n* = 22	63.0 (59.5-66.5)
Lung function test parameters, *n* = 4^e^	
FEV1% predicted	55.00 (52.75-57.25)
FVC % predicted	61.00 (58.00-64.00)
FEV1/FVC	0.90 (0.76-1.04)
Liver function test parameters, *n* = 6^e^	
Alkaline phosphatase (U/liter)	125.50 (83.50-167.50)
Alanine transaminase (U/liter)	16.00 (13.50-18.50)
Aspartate aminotransferase (U/liter)	19.00 (17.00-21.00)
Total bilirubin (mg/dl)	0.44 (0.32-0.56)
Albumin (g/dl)	4.40 (4.20-4.60)
Presence of biliary structure, *n* = 6^e^	3 (50.0%)
Kidney function test parameters, *n* = 1^e^	
Creatinine (mg/dl)	0.96
Estimated glomerular filtration rate (ml/min/1.73 m^2^)	>60

Abbreviations: ACR, acute cellular rejection; FEV, forced expiratory volume; FVC, forced vital capacity; pAMR, pathological antibody-mediated rejection.

Values shown as frequency (percent), median (interquartile range), or total sum with median (interquartile range).

a Exclusion of 2 patients due to deceased before 1-year post-transplant.

b Exclusion of 2 patients due to being transplanted less than one year ago.

c Exclusion of 1 patient due to deceased before 1-year post-transplant.

d Exclusion of 1 patient due to being transplanted less than one year ago.

e Lung function test parameters were assessed only among patients who received lung transplants, liver function test parameters and biliary structure were assessed only among patients who received liver transplants, and kidney function test parameters were assessed only among patients who received kidney transplants.

At 1 year, the median left ventricle ejection fraction was 63.0% (IQR 59.5-66.5). For patients with a lung transplant, the median forced expiratory volume in 1 second (FEV1) at 1 year was 55.0% of predicted (IQR 52.8-57.3) and forced vital capacity (FVC) was 61.0% of predicted (IQR 58.0-64.0). The FEV1/FVC ratio was 0.90 (IQR 0.76-1.04). None of the recipients were noted to have radiographic findings of chronic lung allograft rejection by computed tomography of the chest. For liver transplant patients, the median alkaline phosphatase was 125.50 U/liter (IQR 83.50-167.50), alanine transaminase was 16.00 U/liter (IQR 13.50-18.50), aspartate aminotransferase was 19.00 U/liter (IQR 17.00-21.00), total bilirubin was 0.44 mg/dl (IQR 0.32-0.56), and albumin was 4.40 g/dl (IQR 4.20-4.60). Three (50.0%, *n* = 6) patients who received a liver transplant had biliary strictures at 1 year.

## Discussion

Historically, survival for ACHD patients post-transplant has been limited by many factors. Some are institutional, as centers that perform a low volume of heart transplants annually have worse survival at 1 year compared to high-volume centers.^12^ However, most stem from the complicated physiology of such patients. Traditional markers of severity, such as MCS use, have only recently become used for ACHD patients. This makes it challenging to discern the appropriate “window” in which to refer ACHD patients for transplant. Despite this, timing of evaluation is crucial, and delaying evaluation after Fontan failure is associated with worse long-term outcomes.^13^ At our institution, we refer ACHD patients to the transplant team when there are early signs of decline to allow for longitudinal follow-up. Indications for listing include worsening functional class refractory to reasonable medical interventions, significant bystander organ dysfunction (kidney, liver, or pulmonary vascular disease), and refractory lymphatic complications such as protein-losing enteropathy or plastic bronchitis. We perform yearly cardiopulmonary exercise testing to determine objectively when the functional class begins to decline. All patients are presented at both the multidisciplinary congenital heart disease committee and the heart transplant committee before acceptance for listing.

Of patients with ACHD listed for transplant, Fontan failure is one of the most common indications, and 50.0% of our patients had a previous Fontan procedure. These patients are surgically of high complexity as they often need extensive pulmonary artery reconstruction and have had multiple prior sternotomies.^14^ Additionally, they have an increased risk of perioperative bleeding due to the presence of aortopulmonary and veno-venous collaterals. Recently, many centers have begun aggressive preoperative and postoperative coil embolization of collateral vessels to minimize complications from bleeding in patients with the Fontan procedure.^15^, ^16^

Our approach is to routinely assess the burden of collateral vessels via cross-sectional imaging in all patients with cavopulmonary circulation before transplant, recognizing that this palliative circulation can lead to the formation of these collaterals. Veno-venous collaterals and arteriovenous malformations are not routinely intervened upon pretransplant. We do, however, aim to close all aortopulmonary collaterals (APCs) percutaneously before transplant. To accomplish this, some patients require serial congenital catheterizations due to high APC burden, and in this study, there was a median of 2 catheterizations per Fontan patient before transplant. If left uncorrected, APCs can cause increased volume load to the left ventricle of the transplanted heart and lead to graft dysfunction. After transplant, we perform a repeat computed tomography scan to evaluate for residual APCs and intervene as clinically indicated. Only 2 (13.3%) Fontan patients in this study required embolization post-transplant.

Despite these challenges, our center had a 92.9% 1-year survival for this cohort of ACHD patients. This is consistent with survival rates at other high-volume centers in the United States.^12^, ^17^, ^18^ The 2 deaths we experienced within the first year were both patients who had received a heart-liver transplant for Fontan-associated liver disease (FALD). Despite evidence that patients with FALD may have superior outcomes with a heart-liver transplant compared to heart-alone, our results highlight the challenging nature of caring for these patients and the up-front increased risk and importance of patient selection.^19^ We had no deaths among heart-only or heart-lung transplant recipients in the first year. Interestingly, there were no episodes of heart rejection among any of the dual-organ transplant recipients. The potential immunoprotective effects of heart-liver transplantation have been documented, but there is no evidence to support the same in heart-lung transplantation.^19^, ^20^ Finally, there was no difference in 1-year mortality comparing DCD and DBD donations, which is consistent with our early experience working with DCD heart transplants.^21^

Our group has previously noted the challenges in this patient population pertaining to surgical complexity and the impact of sensitization.^22^ In efforts to minimize the perioperative risk, our center has a protocol that includes collaboration between the adult congenital heart disease and advanced heart failure programs in the management of patients identified as having a current or eventual indication for heart transplant. In addition to a comprehensive evaluation of the cardiovascular system, a full evaluation of other at-risk systems is pursued which includes the pulmonary, renal, hepatic, and in some cases, the lymphatic system. Evidence of extracardiovascular system dysfunction may play a role in the timing of the evaluation for heart transplant and in some cases, multiorgan transplant is indicated.

When evaluating the pulmonary system, pulmonary function tests are often abnormal due to prior surgeries, but that alone may not be a reason for concomitant heart-lung transplant. Unrepaired congenital heart defects, however, may lead to pulmonary vascular disease, and this is a major indication for combined heart-lung transplant at our center. Medical therapies indicated for pulmonary vascular disease, such as pulmonary vasodilators, are used to try to decrease pulmonary vascular resistance and improve symptoms and functional status. However, in the unrepaired patient with pulmonary overcirculation from shunt physiology, this strategy does not alter the need for en-bloc heart-lung transplant. Conversely, in a Glenn or Fontan circulation, pulmonary vascular disease may be responsive to pulmonary vasodilators and heart-only transplant may be safely pursued in some circumstances.

Evaluation of the hepatic system is important in those with Fontan palliation. Routine protocol-driven surveillance for FALD progression to cirrhosis is the standard of care. Our institutional approach includes cross-sectional imaging in the form of magnetic resonance elastography in all patients.^22^ Most patients will undergo liver biopsy unless there is a clear indication that cirrhosis is present or the indication for liver transplantation has already been met. Additional studies to evaluate for the presence of portal hypertension, such as a screening esophagogastroduodenoscopy or diagnostic paracentesis, are done on a case-by-case basis. If there is evidence to support advanced liver disease, which may manifest as biopsy-proven cirrhosis, hepatic malignancy, or complications associated with portal hypertension, combined heart-liver transplant is pursued. Our center’s approach is to perform heart-liver transplant sequentially utilizing machine perfusion for the liver while heart transplant is performed. Due to insufficient evidence to support a superior outcome in heart-liver transplant compared to heart-alone, we do not offer combined heart-liver transplant to a patient with the failing Fontan without evidence of advanced liver disease. We recognize the immunologic benefit of the liver, particularly in those with the Fontan palliation but do not consider the addition of a liver transplant for immunologic indication alone.

This case series highlights the comparable 1-year outcomes in a high-risk patient population with the use of a comprehensive multidisciplinary team. As patients born with complex congenital heart disease continue to enjoy improved early and midterm survival, special attention to this patient population is needed particularly in identifying the right time for transplant referral and their perioperative management.

## Author contributions

**Conceptualization:** Marcus A. Urey, Reice Robinson. **Data curation:** Reice Robinson. **Formal analysis:** Samvel Gaboyan, Reice Robinson, Marcus A. Urey. **Methodology:** Reice Robinson, Samvel Gaboyan, Marcus A. Urey. **Supervision:** Marcus A. Urey. **Writing – original draft:** Reice Robinson, Marcus A. Urey. **Writing – review and editing:** Reice Robinson, Samvel Gaboyan, Laith Alshawabkeh, Ryan Reeves, Eugene Golts, Victor Pretorius, John Nigro, Mark Kearns, Howaida El-Said, Matthew Carazo, Kimberly Hong, Eric Adler, Paul J. Kim, Amanda Topik, Deborah Raleigh, Angela Meier, Sarah Ellis, Ramon Sanchez, Kamyar Afshar, Aarya Kafi, Christine Lin, Gordon Yung, Aleah Brubaker, Gabriel Schnickel, Kristin Mekeel, Jennifer Berumen, Justin Parekh, Pranab Barman, Veeral Ajmera, Yuko Kono, IrineVodkin, Marcus A. Urey.

## Disclosure statement

The authors declare the following financial interests/personal relationships which may be considered as potential competing interests: Paul Kim reports financial support was provided by the National Institutes of Health. Angela Meier reports financial support was provided by the National Institutes of Health. Paul Kim reports a relationship with CareDx Inc. that includes consulting or advisory. Paul Kim reports a relationship with Natera, Inc. that includes consulting or advisory. Howaida El-Said reports a relationship with Merit Medical that includes consulting or advisory. Ryan Reeves reports a relationship with Abbott Vascular Inc. that includes consulting or advisory. The other authors declare that they have no known competing financial interests or personal relationships that could have appeared to influence the work reported in this paper.

This project was partially supported by the 10.13039/100000002National Institutes of Health, Grants UL1TR001442 (Paul Kim), 1KL2TR001444 (Paul Kim), and R01 AI176554 (Angela Meier). The content is solely the responsibility of the authors and does not necessarily represent the official views of the NIH.
